# Size Distribution Measurement of Insoluble Particles in PM_2.5_ Dispersed in Cell Culture Medium Using Aerosolization Techniques

**DOI:** 10.1021/acsomega.5c04217

**Published:** 2025-09-18

**Authors:** Yuki Kuruma, Hiromu Sakurai, Tomoaki Okuda

**Affiliations:** † Department of Applied Chemistry, Faculty of Science and Technology, Keio University, 3-14-1 Hiyoshi, Kohoku-ku, Yokohama, Kanagawa 223-8522, Japan; ‡ National Metrology Institute of Japan (NMIJ), National Institute of Advanced Industrial Science and Technology (AIST), 1-1-1 Umezono, Tsukuba, Ibaraki 305-8563, Japan

## Abstract

The toxicity of PM_2.5_ has attracted interest and has been widely studied because of its adverse health effects. An in vitro assay exposing cultured cells to test particles was used to assess the biochemical response and evaluate PM_2.5_ toxicity. The physicochemical properties of PM_2.5_ suspensions, particularly particle size distribution, are important parameters that require accurate measurement to reliably assess toxicity. However, the conventional dynamic light scattering method has limited size resolution, making it unreliable for measuring the size distribution of particles with a broad size range. In this study, we present a novel method to accurately measure the size distribution of insoluble particles in PM_2.5_ dispersed in a cell culture medium using aerosolization techniques at a submicrometer size range (100 nm to 1000 nm). This method includes sample pretreatment, aerosolization with a spraying device, and size distribution measurements of the aerosolized particles using an electrical mobility analyzing system. Sample pretreatment involves dialysis to remove dissolved nonvolatile components to minimize the formation of residual particles that may interfere with the size distribution measurements of insoluble particles. To validate the method, we measured the size distribution of the monodisperse polystyrene standard particles, including their mixtures, as well as the polydisperse silica standard particles with a known size distribution dispersed in cell culture medium. As a practical application, we demonstrated the measurement of ambient PM_2.5_ samples collected from an urban atmosphere and a highway tunnel. Our method provides a novel approach for evaluating the size distribution of insoluble particles in cell culture medium.

## Graphical abstract

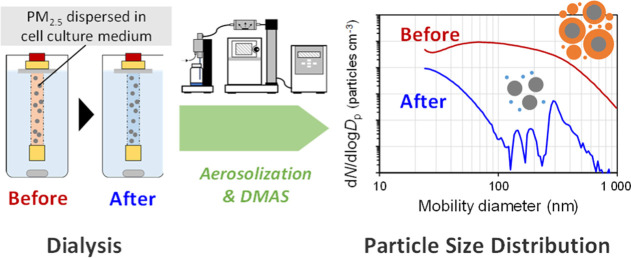

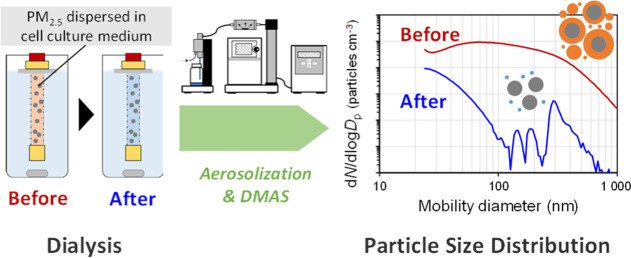

## 1 Introduction

Particulate matter with a diameter smaller than 2.5 μm (PM_2.5_) has attracted interest because of its health effects, and its toxicity has been extensively studied.^1^ Toxicity assessments include two methods: animal exposure experiments (in vivo) and cell exposure experiments (in vitro).^2^ For in vitro assays, the test particles may be added to cultured cells, and the biochemical response of the cells is assessed under controlled conditions. Two methods are used for exposing cells to PM_2.5_ for toxicity assessment: one involves depositing airborne particles directly onto cultured cells, whereas the other involves immersing the particle suspension into the cultured cells. For the latter, airborne PM_2.5_ is collected using various aerosol sampling techniques, including impactors,^3^ filters,^4,5^ and cyclones.^6,7^ The collected PM_2.5_ is suspended in a dispersion medium, such as a cell culture medium or an isotonic solution (e.g., phosphate-buffered saline), to prepare a particle suspension for cell exposure. The cellular biochemical responses include cytotoxicity (cell death), oxidative stress (reactive oxygen species production), inflammatory potential (inflammatory cytokines release), and genotoxicity/mutagenicity. The toxicity of PM_2.5_ is assessed using these indices.

The physicochemical properties of PM_2.5_ suspensions, such as particle size distribution, morphology, surface area, chemical composition, and dispersion stability, are important parameters for evaluating toxicity assessment results^2,8,9^ for in vitro assays. The size distribution of particles insoluble in the cell culture medium is one of the most important parameters of the physicochemical properties.^10,11^ However, in practice, toxicity assessments using in vitro assays are often conducted without accurately evaluating the particle size distribution of PM_2.5_ suspensions. Accurate measurement of the size distribution is challenging because insoluble particles in PM_2.5_ suspensions generally exhibit a broad (polydisperse) size distribution. Commonly used techniques, such as dynamic light scattering (DLS),^12^ lack sufficient size resolution. For DLS measurements of polydisperse particle suspensions, intense light scattering from large particles can obscure weaker light scattering from small particles, making them more difficult to detect. This phenomenon may lead to distortions in particle size distribution. For example, Kato et al.^13^ used DLS to measure the size distribution of particle suspensions containing monodisperse polystyrene latex (PSL) particles with multiple sizes. The results indicated that DLS could not provide a stable, accurate bimodal size distribution for a mixture of particles with a size difference of 3.3-fold or smaller.

Single-particle characterization techniques such as nanoparticle tracking analysis (NTA) and flow cytometry (FCM) offer viable approaches to overcome the limited size resolution of DLS when analyzing polydisperse samples. Unlike DLS, which evaluates fluctuations in scattered-light intensity from an ensemble of particles, NTA and FCM detect and analyze individual particles. This single-particle detection reduces dependence on data-processing algorithms and minimizes the influence of sample polydispersity. Nevertheless, each technique still faces measurement challenges with polydisperse samples. For example, in the submicrometer range, scattering intensity is proportional to the sixth power of particle diameter. Accurate assessment of a broad particle size distribution (e.g., 100 nm–1000 nm) therefore requires detectors with a wide dynamic range. However, commercially available instruments currently lack the required dynamic range, and the methods remain uncommon among general users. Moreover, in NTA, intense light scattering from larger particles hinders the detection of signals from smaller particles, thereby lowering the counting efficiency.^14,15^ In FCM, the scattering intensity depends on each particle’s refractive index, which increases the uncertainty in the measured particle size distribution.^16^ Consequently, few studies have reported the size distribution of polydisperse, compositionally heterogeneous samples such as PM_2.5_. A lack of knowledge regarding the size distribution of a particle suspension can complicate the interpretation of in vitro assay results and lead to unreliable toxicity assessments. The accurate measurement of particle size distributions is necessary to ensure the reliability of in vitro toxicological assessments of PM_2.5_.

The differential mobility analyzing system (DMAS)^17,18^ is an instrument used to measure the size distribution of aerosol particles. DMAS classifies airborne particles (aerosols) by size (more accurately, by electrical mobility) and measures the number concentration of particles for each classified size. It provides a number-based particle size distribution, which enables accurate evaluation of the size distribution of even polydisperse particles. DMAS is frequently referred to by alternative names, such as the Mobility Particle Size Spectrometer or by its trademark name: Scanning Mobility Particle Sizer. In this study, we follow the ISO notation and refer to it as DMAS. Because of its high size resolution, DMAS is widely used for measuring the size distribution of atmospheric aerosols,^19^ industrial nanometal particles,^20,21^ and biological particles.^10,22^ DMAS is a reliable method for particle size distribution measurement and has been adopted for certifying the mean diameter of monodisperse PSL standard particles.^23–25^

To measure the size distribution of the particles dispersed in a liquid phase using DMAS, the particles were aerosolized using sprayers, such as atomizers and nebulizers; however, measuring the size distribution of the particles in a liquid can be challenging under certain conditions. For example, when a particle suspension contains high concentrations of dissolved nonvolatile components in a dispersion medium, spraying with an atomizer or nebulizer can result in the formation of residual particles composed of the dissolved nonvolatile components or the coating of the target insoluble particles with these components, thereby altering particle size distribution.^26^ Because the dispersing media for particle suspensions commonly used in in vitro assays, such as Eagle’s Minimum Essential Medium (MEM) and phosphate-buffered saline, contain a variety of dissolved nonvolatile components (e.g., inorganic salts, amino acids, glucose, and vitamins), spraying the suspension for DMAS measurements can result in the formation of residual particles and coating. Therefore, a purification process,^21,26,27^ including centrifugal filtration and dialysis, is required before the DMAS measurement to remove dissolved nonvolatile components. However, only a few studies have used purification steps to particle suspensions for DMAS measurements, with most previous studies focusing on the measurement of the size distribution of biological particles.^10,22,28,29^ To our knowledge, no studies have examined the size distribution of insoluble particles in PM_2.5_ suspensions using DMAS in vitro.

In this study, we established a protocol for in vitro applications to accurately determine the size distribution of insoluble particles in a culture medium suspension using DMAS. This protocol involves the removal of dissolved nonvolatile components by dialysis to suppress the generation of residual particles and coating. We present size distribution measurements of monodisperse PSL standard particles with known particle sizes and their mixtures, as well as polydisperse silica standard particles with known particle size distribution, to validate the method. As a practical application, we performed a demonstration using ambient PM_2.5_ samples collected from the atmosphere (urban atmosphere or highway tunnel) as test samples.

## 2 Experimental Section

### 2.1 Size Standard Particles

Two types of standard particles were used as test samples to validate the proposed method. The first set consisted of five types of monodisperse PSL standard particles with known mean particle diameters (nominally 100, 200, 300, 500, and 800 nm), purchased from JSR Life Sciences. The mean particle diameters were evaluated by the manufacturer using a transmission electron microscope (Reference values are shown in Table S1). The width of the particle size distribution was within 3% in terms of the coefficient of variation. The stock suspension contained 1% solid by mass in an aqueous medium. Before use, an appropriate amount was taken from the stock suspension and dispersed into MEM (Fujifilm Wako Pure Chemical Corp.) to prepare a particle suspension.

The second set consisted of polydisperse silica standard particles, referred to as Fused Silica Test Particle 0.3–1.5 (FSTP 0.3–1.5),^30^ purchased from the Association of Powder Process Industry and Engineering (APPIE), Japan. Particle size distribution was evaluated by APPIE using a scanning electron microscope.^31^ The certified particle size corresponded to the area-equivalent diameter. Because the particle shape was spherical, the electrical mobility diameter obtained by DMAS measurement should closely match the area-equivalent diameter. According to the certificate of analysis, approximately 80% of the particles fell within a size range of 100 to 440 nm based on the number-based particle size distribution (Reference values are shown in Table S2). As the FSTP 0.3–1.5 sample was supplied as a dry powder form, a test suspension was prepared by dispersing an appropriate amount of the powder into ultrapure water or MEM, which resulted in a mass concentration of 0.1%. Before using the prepared standard particle suspensions, ultrasonic treatment was done for 1 min to ensure redispersion and homogenization.

### 2.2 Ambient PM_2.5_ Particles

Two types of ambient PM_2.5_ samples were used to test the applicability of the method. The first sample consisted of a PM_2.5_ powder collected from the urban atmosphere. This sample was collected from the rooftop of a 22 m high building at Keio University, Yokohama, Japan (35.56°N, 139.66°E), from February 21 to May 16, 2023. The particles were collected using a high-volume aerosol sampler (K-RiC, Keio-Real impactor with Cyclone), which consisted of an impactor and a cyclone.^32^ The K-RiC has lower and upper size limits for particle collection (50% cutoff diameter) of 0.7 and 2.5 μm, respectively. Therefore, the aerodynamic diameter of the collected PM_2.5_ particles ranged from 0.7 to 2.5 μm. The sample was referred to as K-RiC particles.

The second sample consisted of a PM_2.5_ powder collected from inside a highway tunnel. This sample was an environmental certified reference material (NIES No. 8, Vehicle Exhaust Particulates), distributed by the National Institute for Environmental Studies (NIES), Japan.^33,34^ The particles were collected using an electrostatic precipitator in large ventilators connected to a highway tunnel and were primarily composed of automobile emissions. The sample was referred to as Tunnel particles.

As the K-RiC and Tunnel particles were in dry powder form, these samples were dispersed in an appropriate amount of MEM to prepare a PM_2.5_ suspension with a mass concentration of 1 mg mL^–1^. Before using the prepared PM_2.5_ suspensions, ultrasonic treatment was applied for 1 min to ensure redispersion and homogenization.

### 2.3 Dialysis

Dialysis treatment was performed to remove nonvolatile components dissolved in the particle suspension. Ten milliliters of the particle suspension was pipetted into a dialysis tube (diameter: 10 mm, length: 150 mm) with a molecular weight cutoff (MWCO) of 1 × 10^6^ (G235073, Repligen Corp.). Dialysis was initiated by immersing the dialysis tube in 2 L of ultrapure water. The dialysis solution was replaced with fresh ultrapure water every 2 h, and the exchange was repeated four times. The final dialysis step was conducted for 12 to 15 h. Overall, the dialysis process lasted more than 20 h. During dialysis, a magnetic stirrer was used to induce a gentle vortex to facilitate efficient purification. After dialysis, the particle suspension was transferred from the dialysis tube to a separate vial for storage.

### 2.4 Aerosolization and DMAS Measurements

The size distribution of the dialyzed particle suspension was obtained through aerosolization and DMAS measurements. Figure 1 shows the experimental setup for DMAS. The particle suspension was aerosolized using a constant output atomizer (COA) (model 3076, TSI, Inc.). The particle suspension was delivered into the COA at a flow rate of 0.2 mL min^–1^ with a syringe pump (Econoflo, Harvard Apparatus Inc.). Clean air was supplied to the COA at 241 kPa to spray the delivered particle suspension, whereas the downstream side of the COA was maintained at atmospheric pressure. The generated aerosol particles were dried by passing them through a Nafion dryer (Perma Pure LLC) and reducing the relative humidity to below 10%. The aerosol particles were passed through an Am-241 bipolar charge conditioner for electrical neutralization. Particle size distribution was measured using a differential mobility analyzer (DMA) (model 3081, TSI Inc.) and a condensation particle counter (CPC) (model 3010, TSI Inc.). No impactor was used for the DMA to minimize undesired particle loss and removal within the impactor. Instead, the aerosol passing through the charge conditioner was directly introduced into the DMA column. The sample flow rate and sheath flow rate for the DMA were set to 0.15 L min^–1^ and 1.5 L min^–1^, respectively. To compensate for the difference between the sample flow rate of the CPC (1 L min^–1^) and that of the DMA outlet (0.15 L min^–1^), the deficiency (1 L min^–1^–0.15 L min^–1^ = 0.85 L min^–1^) was compensated with the addition of clean air. The control for the DMA and CPC, as well as data acquisition, was done using Aerosol Instrument Manager (AIM) Version 9 (TSI Inc.) software, and the particle size range from 24 nm to 1000 nm was measured. The measurement data obtained from AIM Version 9 were subjected to multiple charge corrections and diffusion loss corrections using AIM Version 10. The measurement system shown in Figure 1 is referred to as the COA-DMAS.

**Figure 1:**
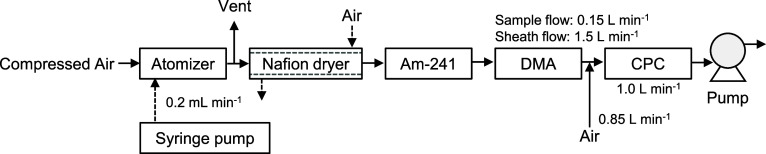
Experimental setup for COA-DMAS.

### 2.5 Optical Particle Counting

Particle size distribution for the PM_2.5_ suspensions before and after the dialysis was measured using an optical particle counter (OPC) for liquid-borne particles to assess changes in size distribution. Three types of OPC sensors, each with a different measurable size range, were used for the measurements. Table S3 shows the specifications of each OPC sensor. Each OPC sensor outputs two types of electrical signals simultaneously, with different gains (amplification factors). The measurable particle size ranges for these two signals overlap in a specific size range. For example, for the KS-28B(200), the two output signals correspond to size distributions of 200 nm–400 nm and 400 nm–2 μm, respectively. Using a multichannel pulse height analyzer to measure both signals simultaneously, a size range of 200 nm–2 μm can be obtained in a single measurement. By sequentially using three types of OPC sensors for a single particle suspension, the size distribution over a range of 200 nm to 100 μm can be obtained. As the OPC sensors are calibrated using PSL particles, the particle size values correspond to the equivalent PSL diameter with the same scattering intensity. In this study, these size values are referred to as the optical diameter. A syringe pump (PSD/6, Hamilton Co.) was used to deliver the particle suspension into the OPC sensors. For acquiring and analyzing the particle signals from the OPC sensors, a high-speed digitizer (PXI-5122, National Instruments, Co.) was used. The digitizer was controlled by a custom-built LabVIEW program, enabling it to function as a pulse height analyzer.

## 3 Results and Discussion

### 3.1 Evaluation of Dialysis Purification

To evaluate the effect of dialysis on particle size distribution, the COA-DMAS measurements were performed before and after the dialysis treatment for a 300 nm PSL particle suspension in MEM medium. Figure S1 shows the particle size distributions measured by COA-DMAS. The number concentration of the 300 nm PSL particle suspension was 1 × 10^9^ particles mL^–1^. Data for the samples with and without dialysis are represented by black and red plots, respectively.

The size distribution obtained from the particle suspension without dialysis showed a high number concentration over the entire size range. No clear peak corresponding to the 300 nm PSL particles was observed because the majority of the particles detected by COA-DMAS were residual particles. The peak corresponding to the 300 nm PSL particles overlapped and was completely masked by these residual particles. The residual particles likely originated from the dissolved nonvolatile components present in the MEM, including inorganic salts (∼1%), amino acids (∼0.1%), and glucose (∼0.1%).

The size distribution obtained from the particle suspension following dialysis showed a primary peak at 300 nm, a secondary peak at 200 nm, a tertiary peak around 150 nm, and a broad hump below 100 nm. These peaks corresponded to the single-, double-, and triple-charged 300 nm PSL particles, respectively. Despite applying multiple charge corrections using AIM software, peaks corresponding to double- and triple-charged particles remained, which were likely due to a deviation between the actual aerosol charge distribution after passing through the bipolar charge conditioner and the distribution assumed in the AIM software, which hindered the complete elimination of these multiple-charged peaks. The particles below 100 nm were residual particles formed through the evaporation and drying of the droplets generated by COA. These droplets contained no 300 nm PSL particles. The presence of a distinct 300 nm PSL particle peak suggests that the dialysis effectively removed the dissolved nonvolatile components from the MEM while preserving the target PSL particles.

The particle recovery rate was evaluated by comparing the number concentration of 300 nm PSL particles before and after dialysis. The particle number concentration was measured using a liquid-borne particle counter (KS-28B(200)) (Table S3). The particle number concentrations before and after dialysis are denoted as *C*_0_ and *C*_1_, respectively, yielding a recovery rate of *C*_1_/*C*_0_ = 0.779. Because nearly 80% of the particles were recovered, dialysis demonstrated minimal particle loss and proved to be an effective pretreatment method for particle suspensions containing a high concentration of dissolved nonvolatile components.

### 3.2 Monodisperse PSL Particles

Validation experiments were performed using monodisperse particles with a known mean particle diameter as test samples. Five PSL particle suspensions of different sizes (100, 200, 300, 500, and 800 nm) and a mixed suspension containing these five PSL particle suspensions were prepared and dialyzed. The particle number concentration of the nonmixed suspensions was 1 × 10^9^ particles mL^–1^, whereas each particle size in the mixed suspensions had a concentration of 0.5 × 10^9^ particles mL^–1^. Figure 2 shows the COA-DMAS measurement results. For all nonmixed suspensions, a unimodal peak corresponding to the PSL particle size was observed, with residual particles appearing below 90 nm. Peaks corresponding to the particle sizes of the five PSL particles were also detected in the mixed suspension. The particle size distribution closely resembled that of the nonmixed suspensions. The heights for the peaks corresponding to each PSL particle were not identical (particularly for the 800 nm particles), which was attributed to the fact that the number concentrations of individual PSL particles in the mixed suspension did not completely match. First, the particle number concentration for the stock suspension had uncertainty, as only the nominal value provided by the manufacturer was available. Second, dilution errors during sample preparation may have contributed to the mismatch in particle number concentrations. Third, systematic errors arising from dialysis treatment and the COA-DMAS measurements may have been contributing factors. In particular, the spraying efficiency of the COA for the 800 nm particles may have decreased. A detailed examination of the sensitivity differences based on particle size was done using polydisperse particles as described in Section 3.3. Overall, the results in Figure 2 indicate that the PSL peaks appeared independently and that the peak particle sizes were consistent with the certified values. While there was some variation in the peak height depending on particle size, the peak heights were generally comparable.

**Figure 2:**
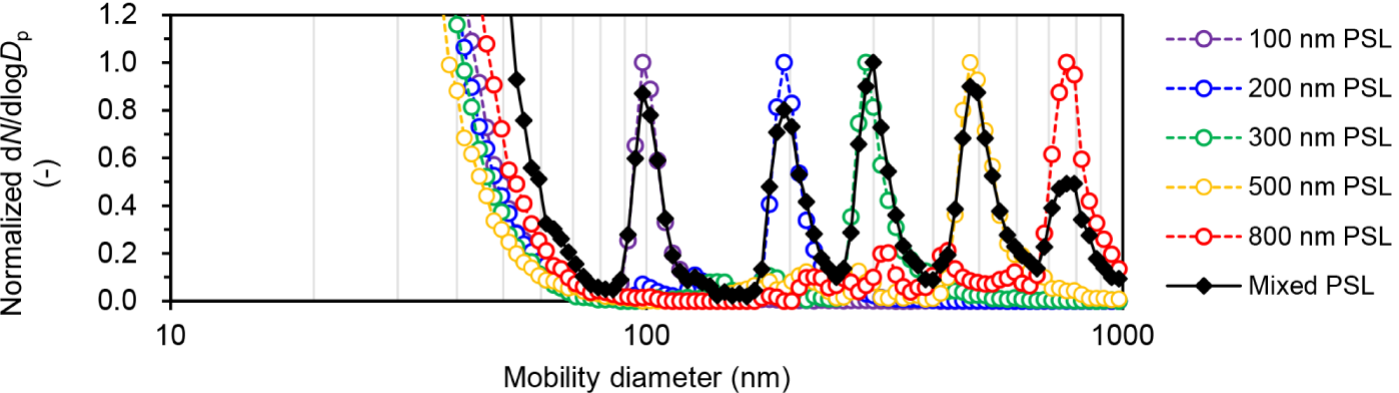
Particle size distributions obtained from the COA-DMAS measurements of unmixed and mixed monodisperse PSL particle suspensions. The vertical axis represents a normalized frequency distribution, where the maximum value of the PSL particle peak is set to 1.

### 3.3 Polydisperse Silica Particles

Validation experiments were performed using polydisperse particles with a known particle size distribution as a test sample. To determine the effect of dialysis treatment on particle size distribution, two types of polydisperse silica particle suspensions with different dispersion media were compared. One suspension used MEM as the dispersion medium (i.e., originally dispersed in MEM and measured by COA-DMAS after dialysis), whereas the other used ultrapure water (i.e., directly dispersed in ultrapure water and measured with the COA-DMAS without dialysis).

Figures 3 and 4 show the COA-DMAS measurement results for the two types of polydisperse silica particle suspensions. Figure 3 shows the frequency distribution, whereas Figure 4 displays the cumulative distribution. Both samples show a peak around 200 nm and exhibit a broad particle size distribution ranging from 70 nm to 1000 nm. In addition, residual particles were observed in the particle size range of less than 50 nm. As shown in Figure 4, the size distribution obtained from the particle suspension with ultrapure water (denoted as UPW) closely matched the reference values, whereas the size distribution obtained from the particle suspension with MEM medium (denoted as MEM and dialysis) was shifted toward smaller sizes by approximately 10 nm.

**Figure 3:**
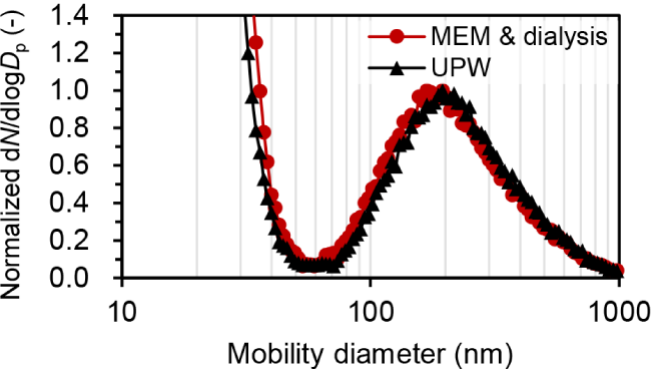
Particle size distributions obtained from the COADMAS measurements of the polydisperse silica particle suspensions. Red circle (MEM and dialysis): Dialyzed silica particle suspension originally dispersed in MEM medium, Black triangle (UPW): Silica particle suspension directly dispersed in ultrapure water. The vertical axis represents a normalized frequency distribution, where the maximum value of the silica particle peak (200 nm) is set to 1.

**Figure 4:**
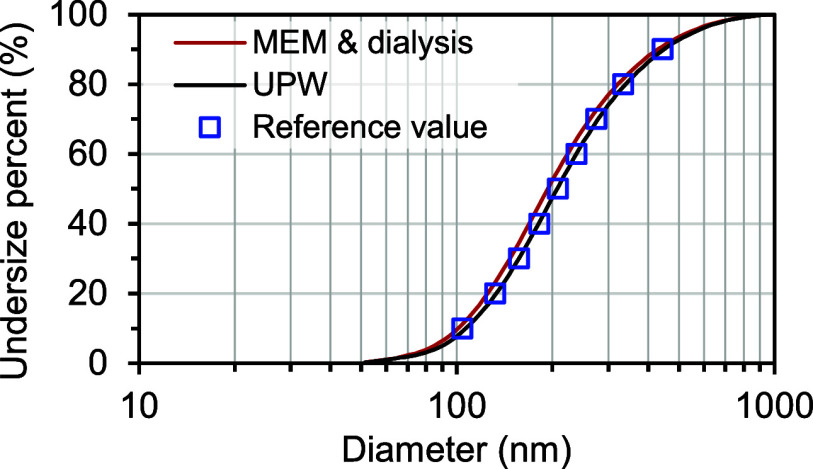
Cumulative frequency curves of particle size distribution obtained from the COA-DMAS measurement of the polydisperse silica particle suspensions. Red (MEM and dialysis): Dialyzed silica particle suspension, Black (UPW): Silica particle suspension directly dispersed in ultrapure water, and blue rectangle: Reference value determined by scanning electron microscopy. Note that the lower size limit was set at 50 nm, and particles larger than 50 nm were included in the count. The vertical axis, labeled “Undersize percent” shows the percentage of particles with a size at or below a given size.

To quantitatively evaluate the particle size distribution obtained from the COA-DMAS measurements, we measured the geometric mean diameter and geometric standard deviation, assuming that the particle size distribution of the polydisperse silica particles followed a log-normal distribution. First, the cumulative distribution (Figure 4) was converted into a log-normal probability plot (Figure S2). The high linearity of the plot (*r* > 0.998) suggests that the size distribution of the polydisperse silica particles follows a log-normal distribution, thus validating this assumption. Table 1 summarizes the results of the geometric mean diameter and geometric standard deviation from Figure S2. The geometric mean diameter (211.3 nm) obtained from the polydisperse silica particle suspension in ultrapure water was comparable with that of the reference value (209.9 nm). The geometric mean diameter (198.9 nm) of the polydisperse silica particle suspension in the MEM media was underestimated by 5.3% (11 nm) compared with the reference value (209.9 nm). As for the geometric standard deviation, an indicator of particle size distribution width, both samples showed values equivalent to that of the reference value (1.75–1.76).

**Table 1:** Geometric Mean Diameters and Geometric Standard Deviations of the Polydisperse Silica Particle Suspensions Calculated from Lognormal Probability Plot (Figure S2)

Sample	Measurement method	Geometric mean diameter (nm)	Geometric standard deviation
MEM medium	Dialysis and COA-DMAS	198.9 ± 1.3^a^	1.76 ± 0.01^a^
UPW medium	COA-DMAS	211.3 ± 0.7^a^	1.76 ± 0.01^a^
Reference value	SEM	209.9	1.75

a Standard deviation for replicate measurements (*n* = 3).

The measurement results from the polydisperse silica particle suspension indicated that dialysis treatment enables accurate particle size distribution analysis using COA-DMAS. As shown in Figure 3, the frequency distribution revealed that the size of the residual particles was similar between the MEM medium sample and the ultrapure water medium sample. This indicated that dialysis was effective at removing the dissolved nonvolatile components from the dispersion medium. As shown in Figure 4, the cumulative distribution revealed that the shape of the particle size distribution in the MEM medium sample and the ultrapure water medium sample was consistent with that of the reference values. The geometric standard deviation closely matched the reference values, indicating that dialysis causes insignificant distortion or broadening of particle size distribution.

On the other hand, the size distribution obtained from the polydisperse silica particle suspension in the MEM medium was slightly shifted. Because the size distribution of the polydisperse silica particle suspension in the ultrapure water medium was consistent with the reference values (see Figure 4), this bias was likely the result of a systematic effect caused by the dialysis process. For example, particle loss may occur during dialysis, depending on particle size. Larger particles may adhere to the bottom of the dialysis tube over prolonged dialysis periods because of their higher gravitational settling velocity, which resulted in their loss. Because of the limited data set, identifying the exact cause is challenging, and further examination of this issue remains a subject for future study. In the case of the polydisperse silica particles, the geometric mean diameter was underestimated by approximately 5.3%. Nevertheless, a reasonable size distribution was obtained. Importantly, when the objective is to semiquantitatively evaluate the size distribution of insoluble particles in PM_2.5_, this level of bias is considered acceptable. Overall, the validation results obtained from the monodisperse PSL particle suspensions (Figure 2) and the polydisperse silica particle suspensions (Figures 3 and 4) indicate that accurate particle size distribution measurements can be achieved through dialysis treatment and COA-DMAS measurements. The lower size determination limit of this method is 50 nm–80 nm, within which the influence of residual particles is negligible (see Figures 2 and 3).

### 3.4 Ambient PM_2.5_ Particles

As a practical application of this method, the size distribution of the insoluble particles in PM_2.5_ suspensions was measured. A sample of K-RiC particles was collected from urban air, whereas Tunnel particles were collected from inside a highway tunnel and were primarily composed of automobile emissions. First, particle suspensions of K-RiC and Tunnel particles were prepared by dispersing them in MEM. These particle suspensions were dialyzed to exchange the dispersion medium from the MEM to ultrapure water. The resulting particle suspensions were measured by COA-DMAS.

Figure 5 shows the measurement results obtained using COA-DMAS for the K-RiC and the Tunnel particle suspensions. Figure 5a,b show the number-based particle size distributions for the K-RiC and the Tunnel particle suspension, respectively, whereas Figure 5c,d show the mass-based particle size distributions, assuming that the particles were spherical with a density of 1.0 g cm^–3^. Moreover, Figure 5 also shows the measurement results obtained by the stepwise variation of the mass concentration of each particle suspension at 1 mg mL^–1^, 0.1 mg mL^–1^, and 0.01 mg mL^–1^ by dilution. The vertical axis represents the particle number concentration, which was corrected by applying the respective dilution factors. The size distribution curves for the diluted suspensions exhibited scatter in the size range above 150 nm, which resulted from the very limited number of particle counts, particularly for the diluted suspensions. For the number-based distribution (Figure 5a,b), both the K-RiC and the Tunnel particle suspensions exhibited a broad peak below 100 nm and exhibited particle size distributions with a long tail extending toward larger particle sizes. Furthermore, as the mass concentration of the particle suspension decreased, both the mode diameter and the tail of the peak at larger particle sizes shifted toward smaller sizes. In the mass-based distribution (Figure 5c,d), both suspensions exhibited a broad peak below 200 nm, while showing another population with a mode diameter of around 1 μm or larger. Similar to the number-based distribution (Figure 5a,b), for the smaller particle population, both the mode diameter and the tail of the peak at larger sizes shifted toward smaller sizes.

**Figure 5:**
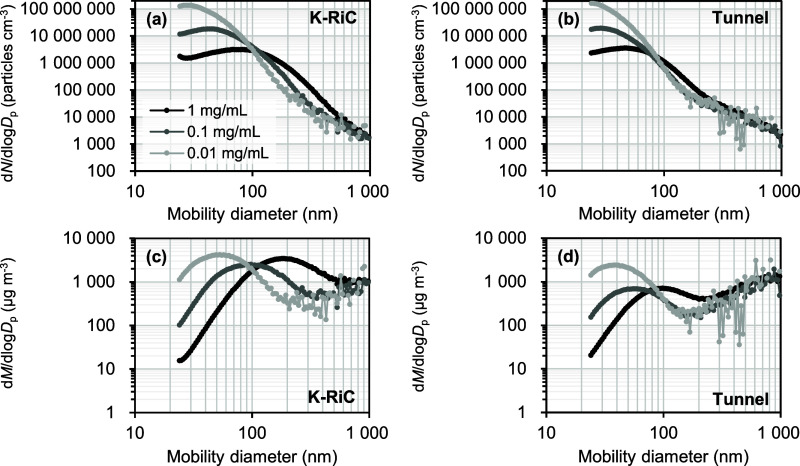
Number based (a, b) and Mass based (c, d) particle size distributions obtained from the COA-DMAS measurement of K-RiC (a, c) and Tunnel (b, d) particle suspensions. Note that the vertical axis represents the particle number/mass concentration after applying a correction by multiplying the dilution factor of each suspension.

The particle size distribution in the 1 mg mL^–1^ suspension, particularly below 100 nm in Figure 5a,b, and below 200 nm in Figure 5c,d, may contain dissolved nonvolatile components as well as insoluble particles, with the latter being the primary measurement target. These dissolved nonvolatile components either originated from the elution of PM_2.5_, were initially present in the dispersing medium (MEM), or both, and remained in the suspension even after dialysis. When these dissolved nonvolatile components remain in the particle suspension, they may form residual particles that do not contain the target particles or coat the surface of the target particles, resulting in an apparent increase in particle size during aerosolization through the COA. Another possibility, which is not associated with the presence of dissolved nonvolatile components after dialysis, is the formation of aggregates consisting of multiple target particles. This occurs when the number concentration of the target particles in the suspension is too high, which results in multiple target particles in a single droplet during aerosolization, causing an overestimation of particle size as well as an underestimation of the number concentration of the target particles. In such cases, accurately determining the true particle size distribution of the insoluble particles in the PM_2.5_ suspension becomes difficult. Therefore, a careful analysis of the resulting particle size distributions is required.

We assumed a situation in which a single droplet generated by an atomizer contains one insoluble particle along with dissolved nonvolatile components. A change in particle size due to coating with dissolved nonvolatile components depends on their concentration in the droplet. When the droplet diameter generated by the atomizer remains constant, the size of the dried particles increases as the concentration of the dissolved nonvolatile components increases. Conversely, as the concentration of the dissolved nonvolatile components decreases, the size of the dried particles decreases. At very low concentrations, the particle size asymptotically approaches the true size of the insoluble particle. With respect to the formation of residual particles composed of dissolved nonvolatile components and free of insoluble particles, their size decreases as the concentration of dissolved nonvolatile components decreases, which results in less overlap with the size of the target insoluble particles (Section 3.1 and Figure S1). Therefore, when dissolved nonvolatile components are present at high concentrations, diluting the suspension can reduce the effect on particle size. The formation of aggregates composed of multiple target insoluble particles can also be suppressed by diluting the suspension, as dilution reduces the probability of producing droplets that contain multiple insoluble particles. Because of the potential effect of the dissolved nonvolatile components and aggregates, one approach to determining the true size distribution of the target insoluble particles is to measure the particle size distribution of a series of diluted samples.

Figure 5 shows the particle size distribution for 1 mg mL^–1^ PM_2.5_ suspensions along with those for the diluted PM_2.5_ suspensions (0.1 mg mL^–1^ and 0.01 mg mL^–1^). As indicated earlier, both the K-RiC (Figure 5a) and the Tunnel particle suspension (Figure 5b) exhibited a trend in which decreasing the mass concentration of the particle suspension shifts the tail on the larger particle size side of the primary peak toward smaller particle sizes. A comparison of the particle size distributions of the K-RiC particle suspension at 0.1 mg mL^–1^ and 0.01 mg mL^–1^, corrected for the dilution factor, revealed an overlap above 200 nm. This suggests that particles larger than 200 nm represent the true size distribution of the insoluble particles, which are unaffected by the dissolved nonvolatile components or aggregates. Similarly, a comparison of the particle size distributions of the Tunnel particle suspension at 0.1 mg mL^–1^ and 0.01 mg mL^–1^ revealed significant overlap above 90 nm, with a sharp gradient change occurring around 150 nm. Particles larger than 150 nm represent the true size distribution of the insoluble particles, which are unaffected by the dissolved nonvolatile components. Likewise, the particle size distribution from 90 to 150 nm corresponded to insoluble particles. The significant difference in particle size distribution across the 150 nm threshold was likely due to the coexistence of multiple particle populations with different origins and size distributions. To determine whether the size distribution curve below 200 nm for the K-RiC particle suspension and below 90 nm for the Tunnel particle suspension represented the true size distribution, further measurements using diluted suspensions are needed. Nevertheless, it is unlikely that the large population of particles below 200 nm for the K-RiC particle suspension and below 90 nm for the Tunnel particle suspension for the most diluted suspensions was solely due to residual particles composed of dissolved nonvolatile components because the size distribution curve of the true size distribution increased toward smaller sizes at these thresholds. In other words, a large number of target insoluble particles were likely present in the size range below 200 nm in the K-RiC particle suspension and below 90 nm in the Tunnel particle suspension. Of note, a large number of K-RiC particles were present in the size range below the lower cutoff of the K-RiC sampler (i.e., 0.7 μm).

To determine whether the observed trend of the true number-based particle size distribution to increase toward smaller sizes for both suspensions was real and not an artifact caused by dialysis, aerosolization, or DMAS measurements, independent OPC measurements were performed to assess particle size distribution in the liquid phase. These measurements were performed using three types of sensors (see Table S3) on the K-RiC and Tunnel particle suspensions, and yielded particle size distributions ranging from 200 nm to 100 μm, with an overlap of 200–1000 nm with the COA-DMAS measurements. A comparison of the particle size distributions before and after dialysis was also conducted.

Figure 6 shows the size distribution for the K-RiC and the Tunnel particle suspensions. The horizontal axis represents the optical diameter, which corresponds to the scattering intensity of the PSL particles, whereas the vertical axis represents the “liquid-borne” particle number concentration. For both samples, the particle size distribution exhibited a long tail beginning at approximately 500 nm and extending toward larger sizes. Particles larger than 2.5 μm in size, up to 40 μm, were detected. For particle sizes below 500 nm (Figure 6a–d), the particle number concentration appeared to plateau or decrease toward smaller sizes, which may be attributed to the underestimation of the particle number concentration resulting from the coincidence losses in the OPC sensors. The plateau or decrease in the concentration at smaller particle sizes observed in the OPC measurements did not contradict the increase in the concentration at smaller particle sizes observed in the DMAS measurements. In the following discussion, the size distribution data from the OPC measurements will be limited to particle sizes of approximately 500 nm and above.

**Figure 6:**
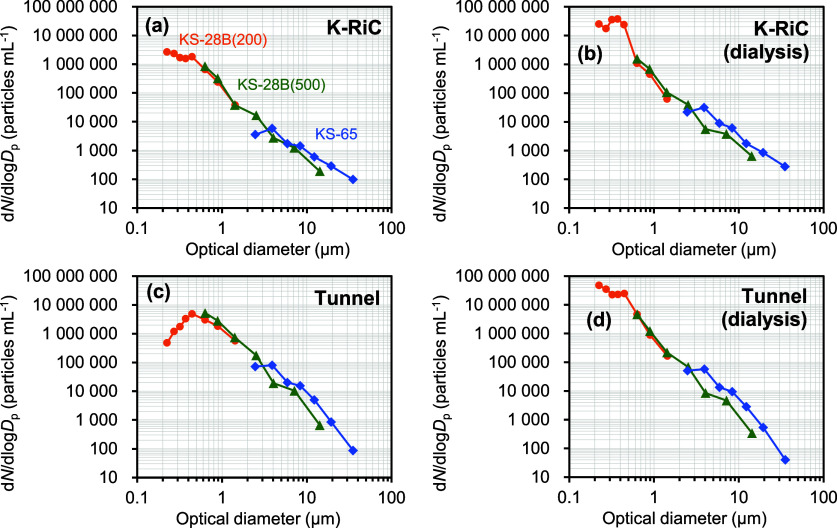
Particle size distributions obtained from the OPC measurement of K-RiC and Tunnel particle suspensions. (a): K-RiC particle suspension before dialysis, (b): K-RiC particle suspension after dialysis, (c) Tunnel particle suspension before dialysis, and (d): Tunnel particle suspension after dialysis. Orange circle: KS-28B(200) sensor, green triangle: KS-28B(500) sensor, and blue diamond: KS-65 sensor.

Both samples before and after dialysis (K-RiC particle suspension: Figure 6a,b; Tunnel particle suspension: Figure 6c,d) exhibited no significant visual differences. For example, for both suspensions, the slope of the long tail beginning at 500 nm and extending up to 40 μm did not appear to change significantly through dialysis. The particle number concentration was generally comparable. The OPC measurement results indicated that the particle size distribution of the PM_2.5_ suspension remained largely unaffected, suggesting that dialysis treatment is effective as a sample pretreatment method for the COA-DMAS measurements.

Particle size distributions for the K-RiC and the Tunnel particle suspensions obtained from the OPC measurements exhibited similar distribution patterns compared with those obtained from the COA-DMAS measurements. The particle size distributions obtained from the OPC and COA-DMAS measurements exhibited a trend in which the particle number concentration increased as the particle size decreased for both suspensions. The particle sizes obtained from the OPC measurements corresponded to the equivalent PSL particle diameter with the same light scattering intensity, whereas those from the COA-DMAS measurements corresponded to the electrical mobility diameter, which resulted in differences in the definition of particle size. Therefore, it is generally inappropriate to directly compare the particle size distributions obtained from the OPC and COA-DMAS measurements. Nevertheless, an attempt was made to perform a quantitative comparison of the particle size distributions.

Figure S3 shows a comparison of the power-law exponents derived from fitting a power-law function to the particle size distributions between the COA-DMAS and OPC measurements. For the COA-DMAS measurements (Figure S3a), the data used for fitting were obtained from the 0.01 mg mL^–1^ suspensions (Figure 5a,b), whereas those for the OPC measurements (Figure S3b) were obtained from the dialyzed suspensions (Figure 6b,d). For the COA-DMAS results, the particle size range for the fitting function was divided into two regions: the smaller particle size range and the larger particle size range (refer to the legend in Figure S3a). Note that the larger particle size range overlapped with that of the OPC measurements. For the K-RiC particle suspension, the power-law exponents obtained from the COA-DMAS (denoted as the orange triangle in Figure S3a) and the OPC measurements (denoted as the orange triangle in Figure S3b) were −2.1 and −2.3, respectively, indicating that they were generally comparable. For the Tunnel particle suspension, the power-law exponents obtained from the COA-DMAS measurements (denoted as the light blue square in Figure S3a) and the OPC measurements (denoted as the light blue square in Figure S3b) were −1.6 and −2.7, respectively, indicating a somewhat larger discrepancy. Figure S3 suggests that although the power-law exponents derived from the fitting are not strictly identical between the COA-DMAS and the OPC measurements, both suspensions exhibited a clear trend in which the particle number concentration decreased as the particle size increased. Specifically, the particle number concentration followed a power-law decay with an exponent ranging from −1.6 to −2.7.

The reason for the difference in the power-law exponent is unclear; however, it may be attributed to factors such as particle nonsphericity or optical properties. For example, in the case of soot particles, which could be a major constituent of the Tunnel particles, nonsphericity increases as the particle size increases. As nonsphericity increases, an overestimation of the mobility-equivalent diameter relative to the volume-equivalent diameter also increases. As a result, the mobility-equivalent diameter distribution extends toward the larger size range compared with the volume-equivalent diameter distribution, potentially leading to a smaller power-law exponent obtained from the power-law fitting. In addition, because the response curve of an optical particle counter (OPC) varies depending on the refractive index of the particles, the difference in light scattering intensity with respect to particle size may also vary. Consequently, the optical-equivalent diameter distribution may become either broader or narrower compared with the volume-equivalent diameter distribution. This may lead to either a decrease or an increase in the power-law exponent. To determine the basis for these differences, including the possibilities mentioned above, a detailed evaluation of the particle characteristics is required, which is beyond the scope of this study.

For the number-based particle size distribution of both particle suspensions, the distribution was concentrated on smaller sizes. Regarding the particle size distribution below 1000 nm obtained from the COA-DMAS measurements, let us assume that the distribution, which appears as a straight line on a log–log plot between 200 nm and 1000 nm, continues along the same line down to 40 nm. Based on this assumption, the ratio of *n* = d*N*/d log *D*_p_ at 200 nm to that at 1000 nm, *n*_200nm_/*n*_1000nm_, is approximately 32 for the K-RiC particles and approximately 14 for the Tunnel particles. Because the Δlog *D*_p_ values for the ranges 200–1000 nm and 40–200 nm are the same, the particle number ratio Δ*N*_40–200nm_/Δ*N*_200–1000nm_, where $\Delta N=\int nd\;\log\;D_{p}$ , is also equal to *n*_200nm_/*n*_1000nm_, resulting in approximately 32 for the K-RiC particles and 14 for the Tunnel particles. For the Tunnel particles, the slope of the size distribution was steeper for the smaller particle size side below approximately 150 nm. Therefore, the ratio Δ*N*_40–200nm_/Δ*N*_200–1000nm_ could be greater than 14. In either case, the results indicate that particles smaller than 200 nm account for greater than 90% of the total particle number concentration. It should be emphasized that this conclusion is based on a bold assumption about particle size distribution below 200 nm. To obtain accurate particle number concentrations, direct measurements are necessary. In particular, to determine the true size distribution below 200 nm for the K-RiC particles and below 90 nm for the Tunnel particles, further dilution of the suspension alone is likely insufficient. Instead, it is necessary to use a sprayer, such as an atomizer specifically designed for generating fine droplets^35^ or an electrospray,^20,22^ which produces smaller droplets than the current COA (nominal droplet size of 0.3 μm in geometric mean diameter, according to the manufacturer’s specifications), to suppress the formation of residual particles, coatings, and aggregates. In addition, the dialysis membrane may need to be replaced with one that has a smaller MWCO, as the membrane used in the present study (MWCO = 1 × 10^6^) may have allowed the target insoluble particles smaller than 100 nm to pass through.

The OPC measurements revealed that, although the particle number concentration was low, particles ranging in size from several micrometers to approximately 40 μm were included in the tail of the peak. This may be attributed to the aggregation of particles in the PM_2.5_ samples, either during the preparation of the particle suspensions or during their collection in the PM_2.5_ samplers (e.g., at the bottom of the cyclone or on the surface of the electrostatic precipitator). Because particles visible to the naked eye were observed in the K-RiC and Tunnel particle suspensions stored in vials, the detection of particles as large as 40 μm did not appear to be an error in the OPC measurements. These micrometer-sized particles had no significant effect on the total number concentration; however, because the mass (or volume) of a spherical particle is proportional to the cube of its diameter, even a small number of larger particles can significantly influence the total mass concentration. For the COA-DMAS measurements, the number-based particle size distributions were converted to mass-based particle size distributions, and the resulting mass-based size distributions of both particle suspensions (Figure 5c,d) indicate the presence of an additional particle population extending beyond 1 μm, along with the population in the submicrometer to nanometer range that probably contained both target insoluble particles and dissolved nonvolatile components, although its tip was not observed within the measurable size range (up to 1 μm). It should be noted that this approach may yield large uncertainties in the mass concentration because of the underlying assumptions and may not be suitable for rigorous evaluation. Specifically, the definition of the particle size is based on the electrical mobility diameter, and the conversion from a number-based to a mass-based distribution depends on several assumptions, including the spherical particle shape and the particle density. Nevertheless, this approach remains valuable for analyzing mass-based particle size distributions given the lack of more suitable measurement techniques. In particular, for in vitro studies, both the number-based and mass-based particle size distributions for the particle suspensions used in the cell exposure experiments provide essential information. Therefore, the ability to evaluate both number- and mass-based size distributions is an advantage for COA-DMAS measurements.

## 4 Conclusion

A novel measurement protocol, which includes the removal of dissolved nonvolatile components by dialysis treatment, aerosolization, and DMAS measurement, was proposed for measuring the size distribution of insoluble particles in PM_2.5_ suspensions for in vitro assays. Our method was validated using monodisperse PSL standard particles and polydisperse silica standard particles. The results indicate the capability of this method to accurately characterize the size distribution of polydisperse particles, which is a challenge for conventional DLS methods. The applicability of this method to various particle size ranges depends on the characteristics of the dialysis and aerosolization devices. In this study, a membrane with a 1 × 10^6^ MWCO was used for dialysis, and the use of the COA atomizer for aerosolization enabled the characterization of the particle size distribution in the range of 100 nm to 1000 nm. In addition, as an application of this method, we performed a demonstration using ambient PM_2.5_ particles as the test sample, suggesting that both suspensions exhibit a number-based distribution profile with a primary peak below 100 nm. For a detailed particle size distribution analysis in this size range, the use of an alternative aerosolization device, distinct from the COA, is required and remains a challenge for future studies. Our method offers a novel approach for determining the particle size distribution for in vitro assays, which is applicable to studies on particle toxicity for not only PM_2.5_, but also other particles, including industrial metal particles and environmental microplastics.

## Supplementary Material



## Notes

COA-DMAS measurement results of the 300 nm PSL particle suspensions before and after dialysis (Figure S1); lognormal probability plot of the particle size distribution of the polydisperse silica particle suspension (Figure S2); curve fittings for the particle size distributions obtained from the COA-DMAS and the OPC measurements (Figure S3); reference values for the monodisperse PSL standard particles and measured mean diameters obtained by the COA-DMAS (Table S1); reference values for the polydisperse silica particles (FSTP 0.3–1.5) taken from the certificate of analysis sheet (Table S2); specification of the OPC sensors (Table S3) (PDF)
