# Correction to “Size Distribution Measurement of Insoluble Particles in PM2.5 Dispersed in Cell Culture Medium Using Aerosolization Techniques”

**DOI:** 10.1021/acsomega.5c10230

**Published:** 2025-10-20

**Authors:** Yuki Kuruma, Hiromu Sakurai, Tomoaki Okuda

We would like to correct the description of the sample specifications in our original publication. The sample referred to as “Tunnel particles” was a sample different from certified reference material (CRM, NIES No. 8). Accordingly, the following passages are replaced.

Section 2.2. Ambient PM_2.5_ Particles, second paragraph:

Error: “The second sample consisted of a PM_2.5_ powder collected from inside a highway tunnel. This sample was an environmental certified reference material (NIES No. 8, Vehicle Exhaust Particulates), distributed by the National Institute for Environmental Studies (NIES), Japan.^33,34^ The particles were collected using an electrostatic precipitator in large ventilators connected to a highway tunnel and were primarily composed of automobile emissions. The sample was referred to as Tunnel particles.”

Correction: “The second sample consisted of tunnel dust collected in Tokyo, Japan, by using a bag filter fixed in the exhaust port of tunnel. As a post-treatment, the collected dust was passed through a 100 μm stainless steel sieve. The particles were primarily derived from automobile emissions and represent one component of PM_2.5_. The sample was referred to as Tunnel particles.”

Section 3.4. Ambient PM_2.5_ Particles, final paragraph:

Error: “(e.g., at the bottom of the cyclone or on the surface of the electrostatic precipitator)”

Correction: “(e.g., at the bottom of the cyclone or on the surface of the bag filter)”

As part of this correction, references 33 and 34 are removed from the reference list.

Additionally, the term “highway tunnel” is replaced with “tunnel” throughout the manuscript.

The CRM is certified for the mass fractions of its constituent elements, but these certified values are not employed in this study and are not the traceability source for any of the results presented. Therefore, the corrections above have no impact on the interpretations or conclusions drawn from the study. We sincerely apologize for the inconvenience caused by these errors.

