# Supplementary material for: Size Distribution Measurement of Insoluble Particles in PM2.5 Dispersed in Cell Culture Medium Using Aerosolization Techniques
Source: ACS Omega. 2025 Sep 18;10(38):43797–807. doi: 10.1021/acsomega.5c04217 (PMC12489708; doi:10.1021/acsomega.5c04217)
Supplement: Supplementary file 1 [file ao5c04217_si_001.pdf]

Supporting Information for Size distribution measurement of insoluble particles in $PM_{2.5}$ dispersed in cell culture medium using aerosolization techniques

Yuki Kuruma[1,2]*, Hiromu Sakurai[2], and Tomoaki Okuda[1]

1. Department of Applied Chemistry, Faculty of Science and Technology, Keio University, 3-14-1 Hiyoshi, Kohoku-ku, Yokohama, Kanagawa, 223-8522, Japan.
2. National Metrology Institute of Japan (NMIJ), National Institute of Advanced Industrial Science and Technology (AIST), 1-1-1 Umezono, Tsukuba, Ibaraki 305-8563, Japan.

Figure S1. Particle size distributions obtained from the COA-DMAS measurements of the monodisperse 300 nm PSL particle suspensions. Red circle: 300 nm PSL particle suspension before dialysis, Black triangle: 300 nm PSL particle suspension after dialysis.

Figure S2. Lognormal probability plot of particle size distribution of the polydisperse silica particle suspension. Red cross (MEM & dialysis): Dialyzed silica particle suspension, Black cross (UPW): Silica particle suspension directly dispersed in ultrapure water, and blue rectangle: Reference value determined by scanning electron microscopy.

Figure S3. Curve fittings for the particle size distributions obtained from (a) COA-DMAS and (b) OPC measurements. The data used for fitting the COA-DMAS and the OPC measurements were taken from the 0.01 mg mL^{-1} $PM_{2.5}$ suspension and the dialyzed suspensions, respectively.

Table S1. Mean diameters of the monodisperse PSL standard particles taken from the certification sheet provided by the manufacturer and measured mean diameters obtained by the COA-MDAS.

Table S2. Cumulative size distribution of the polydisperse silica particles (FSTP 0.3-1.5) taken from the certificate of analysis sheet provided by the manufacturer.

Table S3. Specification of the OPC sensors.

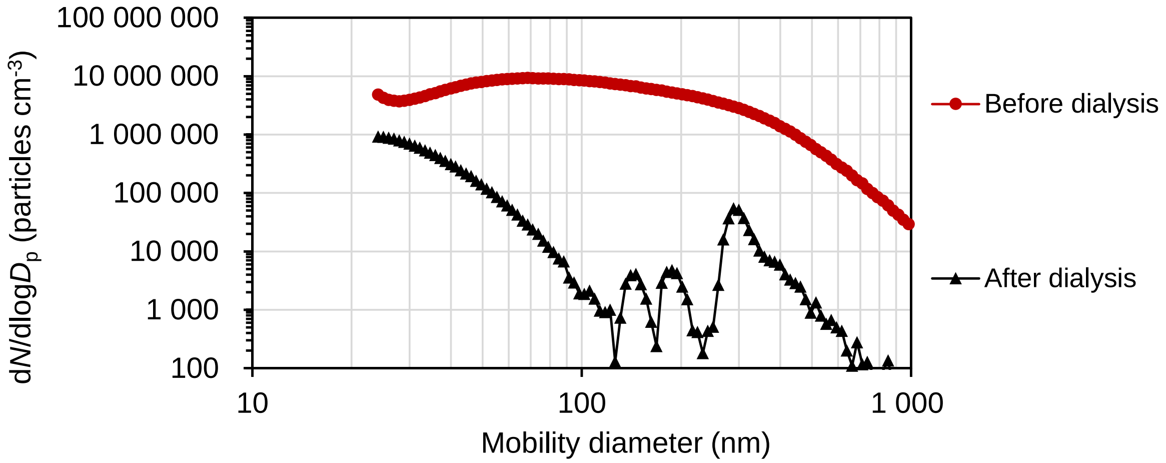

Figure S1. Particle size distributions obtained from the COA-DMAS measurements of the monodisperse 300 nm PSL particle suspensions. Red circle: 300 nm PSL particle suspension before dialysis, Black triangle: 300 nm PSL particle suspension after dialysis.

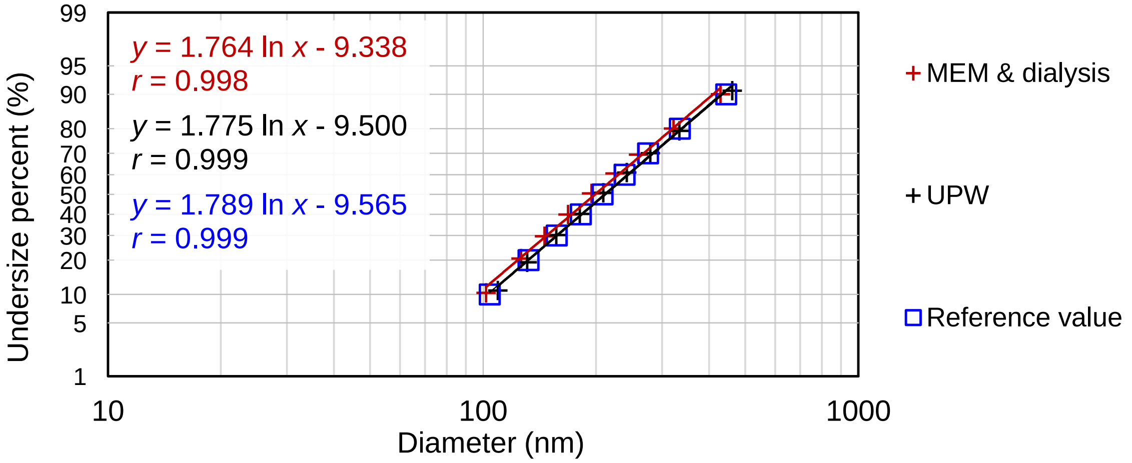

Figure S2. Lognormal probability plot of particle size distribution of the polydisperse silica particle suspension. Red cross (MEM & dialysis): Dialyzed silica particle suspension, Black cross (UPW): Silica particle suspension directly dispersed in ultrapure water, and blue rectangle: Reference value determined by scanning electron microscopy.

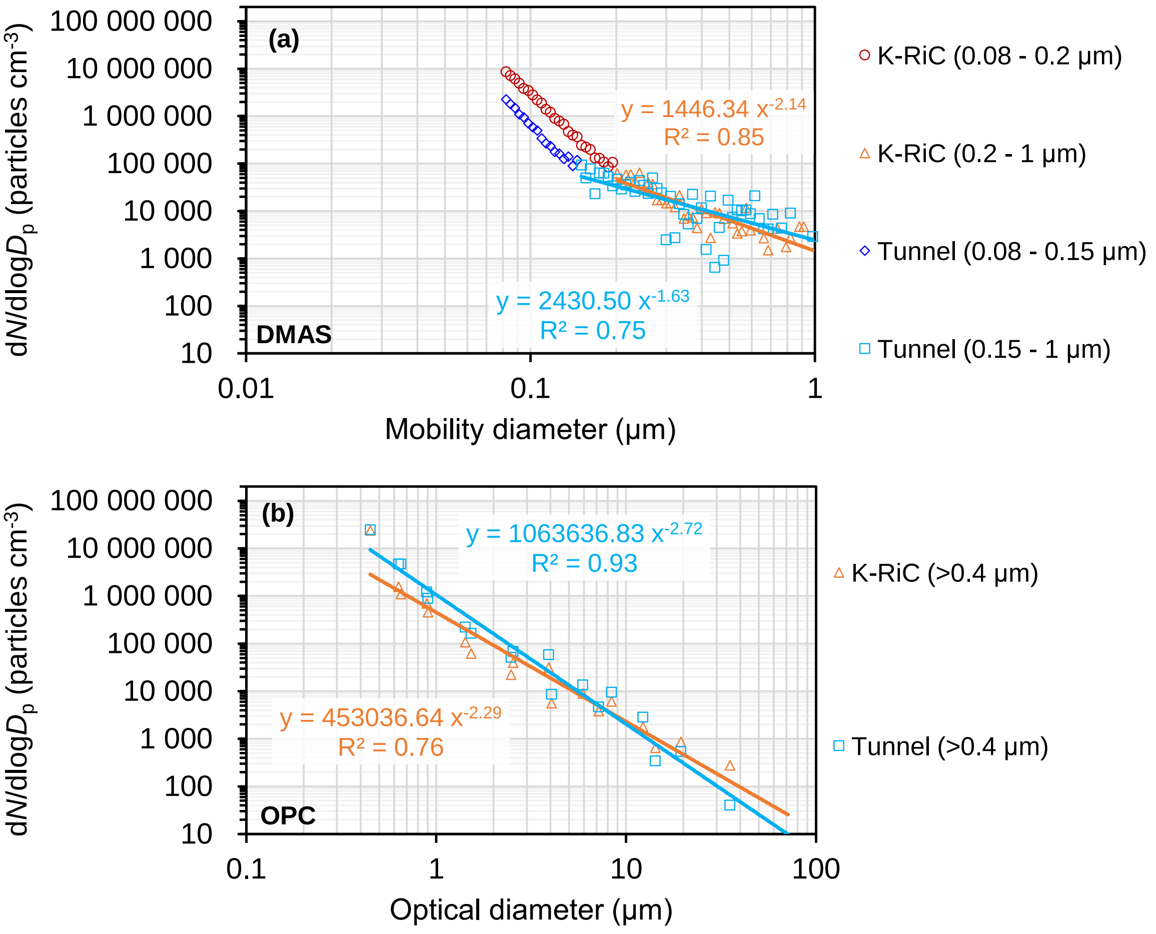

Figure S3. Curve fittings for the particle size distributions obtained from (a) COA-DMAS and (b) OPC measurements. The data used for fitting the COA-DMAS and the OPC measurements were taken from the 0.01 mg mL^{-1} $PM_{2.5}$ suspension and the dialyzed suspensions, respectively.

Table S1 Mean diameters of the monodisperse PSL standard particles taken from the certification sheet provided by the manufacturer and measured mean diameters obtained by the COA-MDAS.

Nominal diameter (nm)	Reference value ± U^* (nm)	COA-DMAS (nm), n = 3
100	100 ± 3	106.0
200	202 ± 5	206.8
300	309 ± 9	316.9
500	506 ± 12	524.2
800	814 ± 19	785.8

$^*U$: Expanded uncertainty (k = 2)

Note: The COA-DMAS setup used in this study measures the mean particle diameter with an accuracy within 6 %. Sources of measurement uncertainty include errors in the DMA sheath flow rate, the incomplete elimination of the multiple-charged peaks, and the presence of peak broadening toward larger sizes for each particle peak (see Figure 2). For the 800 nm PSL standard particles, which are near the upper limit of the measurable size range, no particles with diameters above 1 000 nm are measured.

Table S2 Cumulative size distribution of the polydisperse silica particles (FSTP 0.3-1.5) taken from the certificate of analysis sheet provided by the manufacturer.

Undersize percent* (%)	Diameter (nm)
10	104
20	132
30	157
40	182
50	208
60	238
70	275
80	334
90	444

*The percentage of particles with a size at or below a given size.

Table S3. Specification of the OPC sensors

Model	Type	Measurable size range**	Flow rate	Duration	Manufacturer
KS-28B(200)*	Light scattering	200 nm – 400 nm<br>400 nm – 2 μm	10 mL min^{-1}	30 s	RION Co., Ltd.
KS-28B(500)*	Light scattering	500 nm – 2 μm<br>2 μm – 20 μm	10 mL min^{-1}	30 s	RION Co., Ltd.
KS-65	Light extinction	2 μm – 25 μm<br>25 μm – 100 μm	25 mL min^{-1}	12 s	RION Co., Ltd.

*The manufacturer's official model designation is 'KS-28B', but for identification purposes, it is denoted here as 'KS-28B(200)' and 'KS-28B(500)'.

**OPC sensors output two types of signals with different gain levels. Each signal has different measurable particle size range.
